# Identification and validation of a prognostic index based on a metabolic-genomic landscape analysis of ovarian cancer

**DOI:** 10.1042/BSR20201937

**Published:** 2020-09-28

**Authors:** Qun-feng Zhang, Yu-kun Li, Chang-ye Chen, Xiao-di Zhang, Lu Cao, Fei-fei Quan, Xin Zeng, Juan Wang, Jue Liu

**Affiliations:** 1 Department of Obstetrics and Gynecology, The Second Affiliated Hospital of University of South China, Hengyang, Hunan 421001, P.R. China; 2 Department of Histology and Embryology, Clinical Anatomy and Reproductive Medicine Application Institute, University of South China, Hengyang, Hunan 421001, P.R. China; 3 Hunan Province Key Laboratory of Tumor Cellular and Molecular Pathology, Cancer Research Institute, University of South China, Hengyang, Hunan 421001, P.R. China; 4 Department of Obstetrics and Gynecology, Shenzhen Second People’s Hospital, Shenzhen, Guangdong 518000, P.R. China; 5 Department of Pathology, Huizhou Sixth People’s Hospital, Huizhou, Guangdong 516000, P.R. China; 6 Department of Obstetrics and Gynecology, The First Affiliated Hospital of University of South China, Hengyang, Hunan 421001, P.R. China; 7 Department of Obstetrics and Gynecology, Foshan First People’s Hospital, Foshan, Guangdong 528000, P.R. China

**Keywords:** metabolic-genomic landscape, ovarian cancer, personalized medicine, prognostic index, the cancer genome atlas

## Abstract

**Purpose:** Tumour metabolism has become a novel factor targeted by personalised cancer drugs. This research evaluated the prognostic significance of metabolism-related genes (MRGs) in ovarian serous cystadenocarcinoma (OSC).

**Methods:** MRGs in 379 women surviving OSC were obtained using The Cancer Genome Atlas (TCGA) database. Then, several biomedical computational algorithms were employed to identify eight hub prognostic MRGs that were significantly relevant to OSC survival. These eight genes have important clinical significance and prognostic value in OSC. Subsequently, a prognostic index was constructed. Drug sensitivity analysis was used to screen the key genes in eight MRGs. Immunohistochemistry (IHC) staining confirmed the expression levels of key genes and their correlations with clinical parameters and prognosis for patients.

**Results:** A total of 701 differentially expressed MRGs were confirmed in women with OSC by the TCGA database. The random walking with restart (RWR) algorithm and the univariate Cox and lasso regression analyses indicated a prognostic signature based on eight MRGs (i.e., ENPP1, FH, CYP2E1, HPGDS, ADCY9, NDUFA5, ADH1B and PYGB), which performed moderately well in prognostic predictions. Drug sensitivity analysis indicated that PYGB played a key role in the progression of OSC. Also, IHC staining confirmed that PYGB has a close correlation with clinical parameters and poor prognosis in OSC.

**Conclusion:** The results of the present study may help to establish a foundation for future research attempting to predict the prognosis of OSC patients and to characterise OSC metabolism.

## Introduction

Ovarian cancer is the deadliest malignant tumour of the female reproductive system. Ovarian cancer is the eighth most common cause of lethal malignancies in gynaecology and has a cancer-correlated occurrence rate worldwide, leading to a large social and economic burden [1]. Ovarian serous cystadenocarcinoma (OSC), the most frequent histological type of ovarian cancer, accounts for approximately 75–80% of all ovarian cancer cases [2]. These types of cancer are either symptomless or show similar symptoms to other benign gynaecological diseases until the neoplasm invades the peritoneal surface and is subsequently diagnosed. Most patients with OSC are often diagnosed at an advanced stage. For patients with locally advanced or distant metastatic OSC, existing treatment options are inadequate. Hence, OSC patients have a poor overall survival rate (OS), and the 5-year relative survival rate of OSC is only 45% [3]. Therefore, the identification and development of potential prognostic markers to predict OSC outcomes have high clinical value.

The metabolism of tumour cells is different from that of normal cells, which induces them to maintain a large capacity for proliferation and anti-apoptosis [4]. Tumour metabolism has become a novel driver of personalised cancer drugs, as tumours actively attempt to regulate the metabolic system to extend their lifespans. In recent years, metabolic therapies have been used as therapeutic regimens for multiple cancer types. To date, many researchers have found that anti-metabolic agents could promote the effective elimination of ovarian cancer cells. In addition, Kanakkanthara et al. found that the repression of energy metabolism may be an alternative means of selectively targeting BRCA1-deficient OSCs, which is represented by BRCA1 loss and nicotinamide N-methyltransferase overexpression [5]. Zhang et al. indicated that ACTL6A expression promotes the development and progression of ovarian cancer, especially in the glucose metabolism of cancer cells [6]. Targeting metabolising genes that are specific for ovarian cancer but not homologous normal cells may lead to the successful development of anti-metabolites [7]. Accumulation of glycogen is commonly observed in epithelial ovarian cancer, resulting in chemoresistance [8]. Fatty acid metabolism is also disordered in cancer. Unsaturated lipids are enhanced in ovarian cancer [9,10], which mediate cancer development and progression, especially stemness [9]. The results of these studies indicate that specific metabolic phenotypes increase chemoresistance in ovarian cancer. Furthermore, these findings also suggest that cancer cells’ ability to quickly switch between metabolic substrates and molecular pathways is correlated with phenomena associated with adverse prognosis, such as migration and invasion [11].

The purpose of the present study was to determine the potential clinical value and academic significance of metabolism-related genes (MRGs) in prognostic stratification, as well as the potential of MRGs as markers for targeted therapy of OSC. Subsequently, we analysed the expression of MRGs in combination with clinical characteristics and determined the progression-free intervals (PFIs) of OSC patients by computational methods. We systematically integrated the prognosis and expression profiles of MRGs and developed personalised prognostic features for OSC patients. The potential regulatory mechanisms were elucidated by bioinformatics analyses. The present study may help to establish a foundation for further intensive metabolism-related research with strong potential for progress towards achieving individualised treatment of OSC.

## Methods

### Data extraction

The Cancer Genome Atlas (TCGA) database (https://portal.gdc.cancer.gov/) is the largest cancer gene information database, and it includes data concerning gene expression, miRNA expression, copy number variation, DNA methylation, SNPS and other data. We extracted data for 374 cases of patients with OSC. The characteristics of the population were shown in Supplementary data (clinical-TCGA). Moreover, we downloaded level three FPKM data for subsequent analysis. The transcriptome RNA-sequencing and clinical information of 88 normal ovarian samples were extracted from the GTEX database (https://www.gtexportal.org/). Seventy MRG sets were obtained from the gene set database of the Kyoto Encyclopedia of Genes and Genomes (KEGG) pathways, which included 186 gene sets in total, at the gene set enrichment analysis (GSEA) website (http://software.broadinstitute.org/gsea/downloads.jsp#msigdb). A total of 1466 genes related to human metabolism were included. The method of gene set acquisition was the same as that described above.

### Functional enrichment analyses

The R package ‘ClusterProfiler’ was used for functional annotation of differentially expressed metabolic genes to comprehensively explore the functional correlation of these differentially expressed genes. Gene ontology (GO) and the KEGG were used to assess the relevant functional categories. The GO and KEGG analyses with *P-* and q-values less than 0.05 were considered to be significant.

### Establishing prognostic indicators based on MRGs

The String database was used to construct the network map of metabolic genes. Scores represented each gene action relationship score. We selected ACOT7, CERK, EHMT2, MTAP and PDE8A as the initial genes in the random walking with restart (RWR) algorithm, which has been determined by the ovarian cancer-related pathway in the GSEA database. The random walk model iterated 1e+5 times. The top 200 metabolic genes were selected for subsequent model construction. Univariate Cox analysis was used to select genes associated with prognosis and was used to further construct the prognostic correlation model. After incorporating the expression values of each specific gene, a risk score formula was constructed for each patient. According to the risk score formula, the patients were divided into a low-risk group and a high-risk group, with the median risk score serving as the cut-off point. Survival differences between the two groups were assessed by Kaplan–Meier analysis and compared using log-rank statistical methods. Finally, receiver operating characteristic (ROC) curves were used to study the accuracy of model prediction.

### RWR method

The RWR is a classical sorting algorithm that simulates the random walk on a constructed network from one or more seed nodes. In the process of migration, possible new nodes were identified and ranked from high to low probability. The algorithm is often used to find new disease genes or other related problems. In the present study, we selected five ovarian cancer-related genes (i.e., *ACOT7, CERK, EHMT2, MTAP* and *PDE8A*) as seed nodes, which had been repeatedly verified. The initial probability P^0^ of each seed node was set to 1/T (where T was the number of seed nodes), while the initial probability P^0^ of the non-seed node was set to 0. The RWR simulated the events when these five genes moved across the network. P^l^ was a vector representing the probability of each node after the l-th movement was completed: P^(l+1)^ = (1 − b)YP^l^ + bP^0^. Y is the normalised connection matrix of the moving network. This method can be employed to obtain the interaction information between the constructed gene networks and prioritise the candidate nodes according to the distance between the candidate nodes and the known disease seed nodes. The higher the distance to the seed, the higher the score of the candidate node.

### Clinical samples

A total of 60 OC tissues and 26 normal ovarian tissues were surgically resected in the Huizhou Sixth People’s Hospital (Huizhou, Guangdong, China), The First Affiliated Hospital, University of South China (Hengyang, Hunan, China), and The Second Affiliated Hospital, University of South China (Hengyang, Hunan, China) from 2015 to 2020. The collection and use of tissues were performed in keeping with the ethics standards as formulated in the Helsinki Declaration. Written informed consent was obtained from each patient, which was approved by the research ethics committee of the University of South China. The tissues were made into three pieces of 10 × 10 chips. None of the patients received radiotherapy or chemotherapy.

### Immunohistochemistry staining and scoring

Immunohistochemistry (IHC) was performed with a two-step detection kit (ZSBiO PV73 9000, China). The paraffin-embedded tissue sections were dewaxed in xylene, rehydrated in a graded alcohol system and boiled in a high-pressure autoclaved citric acid buffer (pH 6.0) for 15 min, and peroxidase activity was quenched with 3% hydrogen peroxide for 20 min to avoid nonspecific staining. The sections were washed three times with PBS followed by incubation overnight with anti-PYGB antibody (Abcam, ab251810 at 1/1000 dilution) at 4°C. After that step, the sections were washed with PBS three times and incubated at room temperature for approximately 20 min with a reaction enhancer kit. This step was followed by three washes in PBS, incubation with secondary antibody at room temperature for 20 min, and staining with 3,3-diaminobenzidine (DAB; Zhongshan Biotech, Beijing, China). The sections were dehydrated and sealed after redyeing with Haematoxylin.

Two experienced pathologists independently assessed the percentage of positive cancer cells and their staining strength. The IHC staining intensity was scored from 0 to 2 (0, no staining; 1, weak staining; 2, strong staining). The staining extent was scored from 0 to 4 based on the percentage of immune-reactive cancer cells (0, 1–5, 5–25, 25–75, >75%). A score ranging from 0 to 8 was calculated by multiplying the staining extent score by the intensity score, resulting in negative (0–4) staining or positive (5–8) staining for each example.

### Identification based on the Human Protein Atlas database

The expression of PYGB was analysed using the Human Protein Atlas (HPA) database (https://www.proteinatlas.org/) [12]. Twelve OC tissues and three healthy ovary controls were retrieved. The IHC staining intensity in the HPA database refers to the above scoring method.

### Statistical analysis

The survival curves were generated by the Kaplan–Meier method and compared by the log-rank test. All statistical analyses were performed in the R Programming Language (version 3.6). All statistical tests were bilateral, and *P*<0.05 was considered to be significant.

## Results

### Identification of differentially expressed MRGs

**Figure 1:**
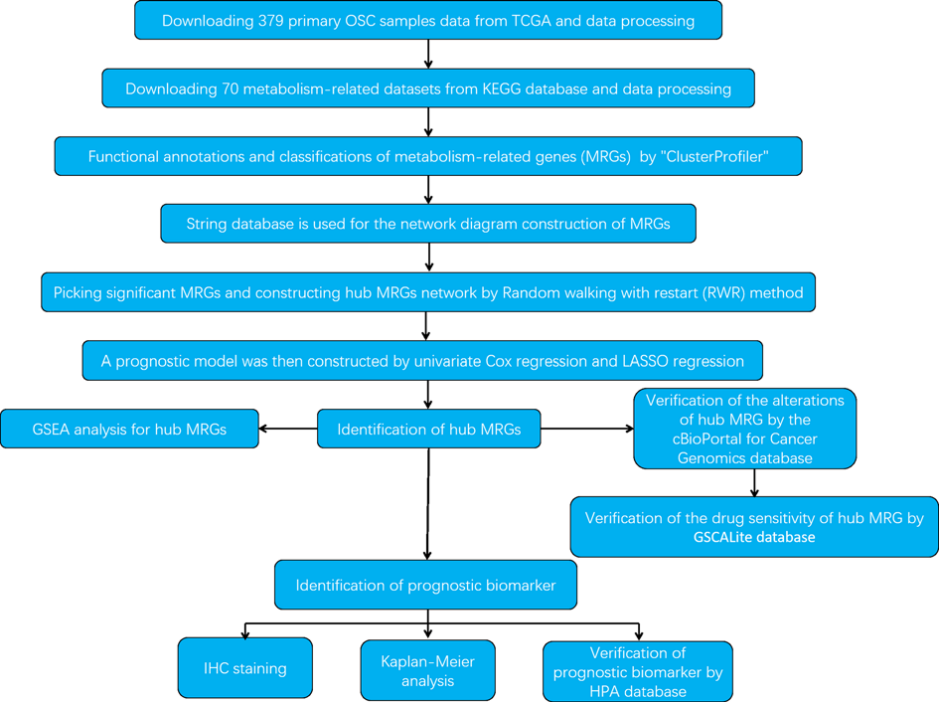
Workflow of the study

The research strategy is presented in Figure 1.

**Figure 2:**
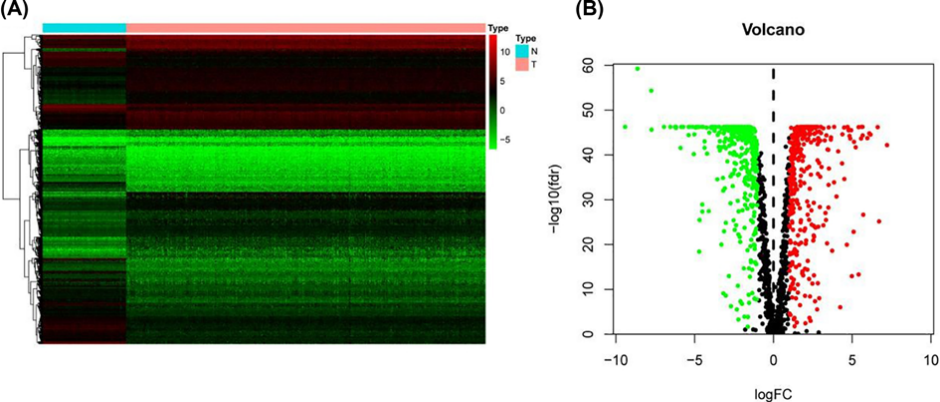
Differential expression of MRGs in OSC and normal ovarian tissues (**A**) Heat map of these MRG expression profiles. (**B**) TCGA data portal for the volcano plot of 701 targets. Green means low level and red means high level.

**Figure 3:**
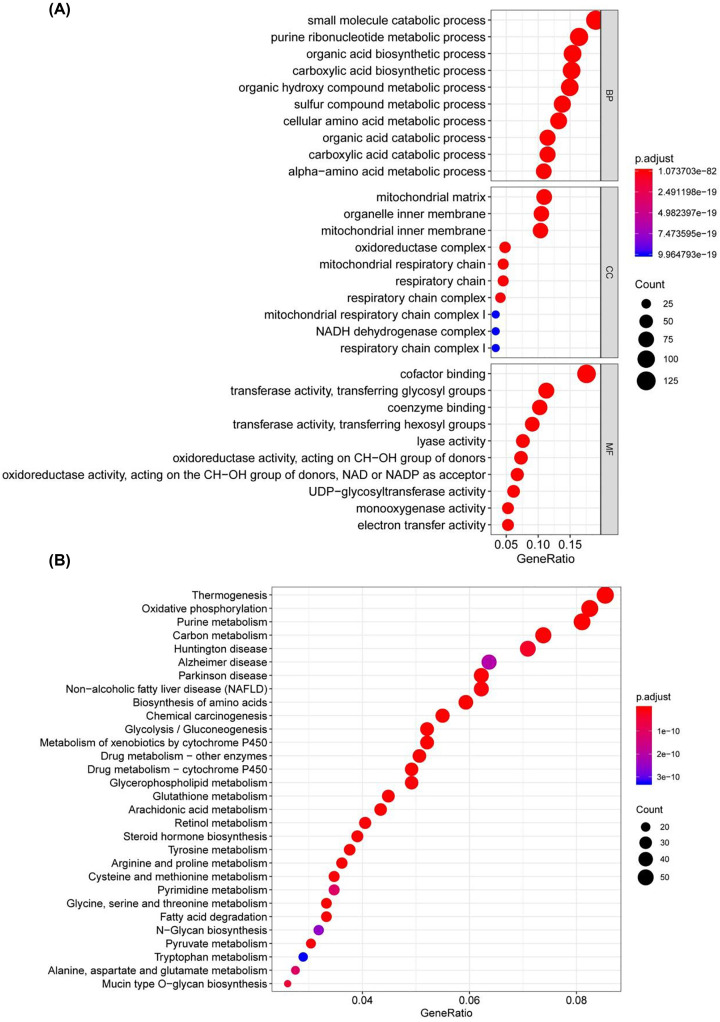
GO analysis of the MRGs (**A**) GO analysis. (**B**) KEGG enrichment analysis.

First, the RNA-Seq data for 379 OSC tissue samples and 88 normal ovarian samples were downloaded from TCGA and GTEX, respectively. The expression values of 701 MRGs were extracted. Considering the indicators of *P*<0.05 and |log2 (Fold Change)| > 1, we obtained 351 up-regulated MRGs and 350 down-regulated MRGs (Figure 2). As we expected, GO enrichment analysis showed that metabolic pathways were most commonly influenced. ‘Small molecule catabolic process’, ‘mitochondrial matrix’ and ‘cofactor binding’ were the most common biological terms for biological processes, cellular components and molecular functions, respectively (Figure 3A). Thermogenesis was the process most frequently enriched by these MRGs in KEGG analysis (Figure 3B).

### Construction of the OSC MRG network

**Figure 4:**
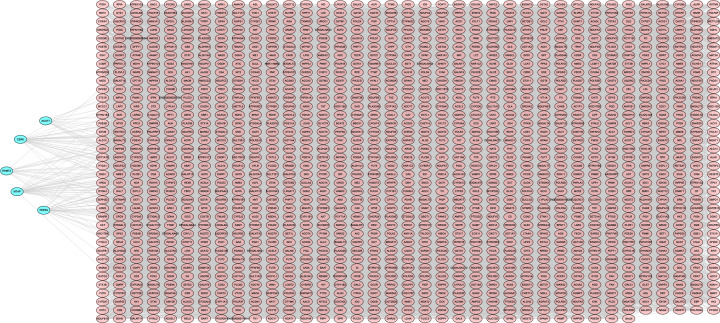
Random walk model within OSC The red and blue nodes represent MRGs and initial genes, respectively. The grey lines show the interactions among these genes.

RWR was used to select these metabolic genes that are related to the pathogenesis of ovarian cancer. All MRGs were included in the String database to build a network of interactions among these genes. As the functional relationship score among genes, scores were used for the network construction of the subsequent random walk model. On the basis of the abovementioned network, the known ovarian cancer pathogenic genes were selected through the KEGG database as the initial genes of the RWR network, that is, ACOT7, CERK, EHMT2, MTAP and PDE8A (Figure 4). The RWR model iterated 100000 times in total. According to the score of the model, the genes with the TOP 200 score value (TOP 200 metabolic genes that can be considered the most closely related to the pathogenesis of ovarian cancer) were selected as the basis for the subsequent model construction.

### Evaluation of clinical outcomes

**Figure 5:**
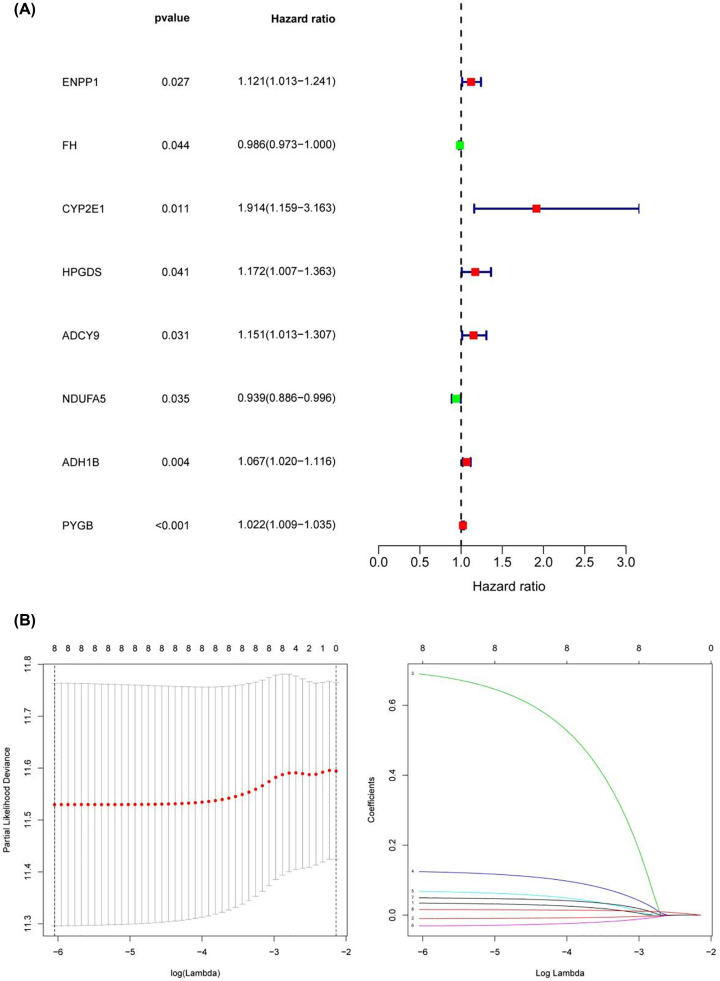
Hub MRG prognostic values (**A**) Prognostic values of genes shown by forest plot of hazard ratios. (**B**) Construction of prognostic signatures based on lasso Cox analysis.

The univariate Cox method was used to analyse the relationship between the above 200 genes and prognosis. A total of eight genes were closely related to the prognosis of ovarian cancer patients, namely, ENPP1, FH, CYP2E1, HPGDS, ADCY9, NDUFA5, ADH1B and PYGB (*P*<0.05) (Figure 5A). Furthermore, lasso regression was used to construct the prognostic model, and the risk score formula was as follows: Risk scores = ENPP1*0.0347 + FH*(−0.010) + CYP2E1*0.690 + HPGDS * 0.124 + ADCY9 * 0.068 + NDUFA5 * (−0.031) + ADH1B * 0.049 + PYGB * 0.016. According to the risk score formula, the patients were divided into a low-risk group and a high-risk group, with the median risk score serving as the cut-off point (Figure 5B).

**Figure 6:**
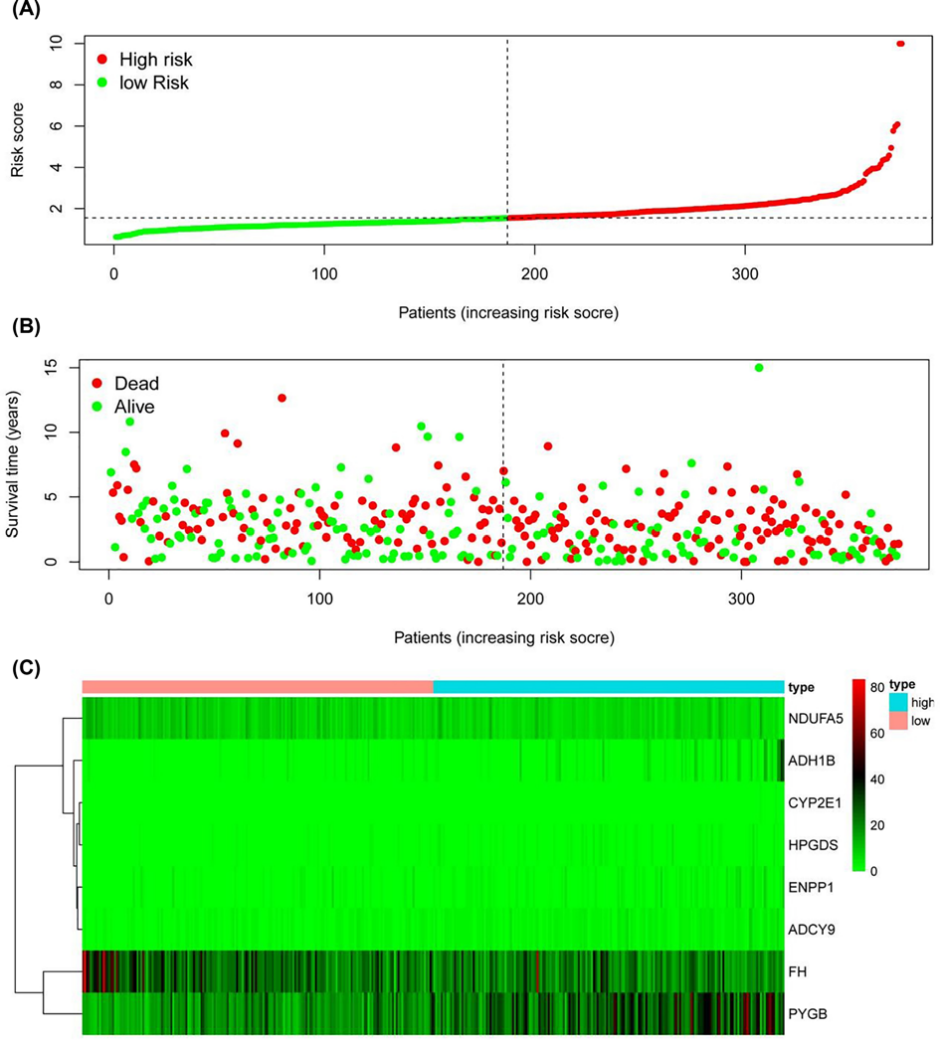
Metabolism-based prognostic index of OSC patients (**A**) The training dataset shows the PI distribution of patients. (**B**) Total survival of patients in the TCGA dataset. (**C**) Heat map of eight hub gene levels extracted by TCGA dataset.

**Figure 7:**
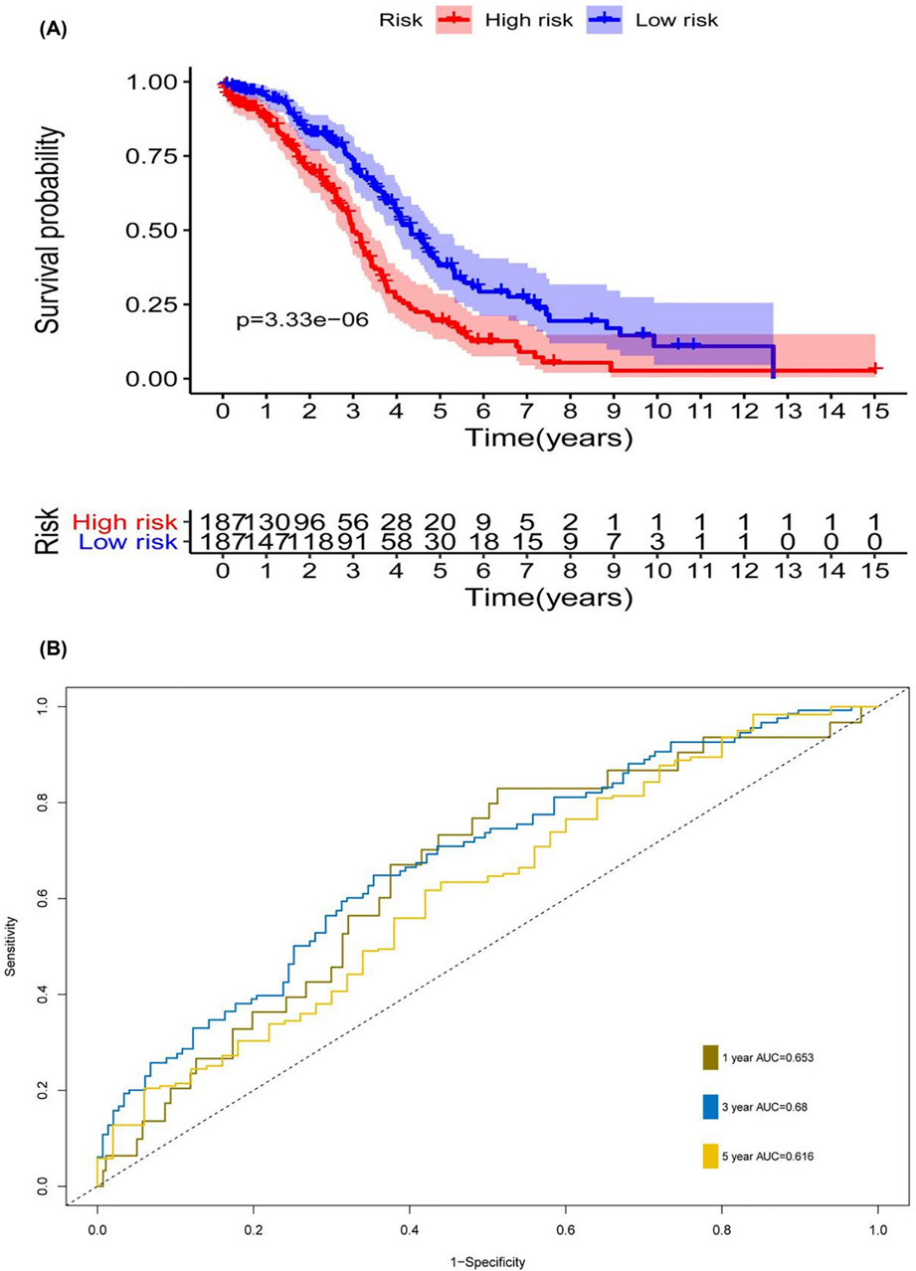
Metabolism-based prognostic index in OSC patients (**A**) TCGA database showed OS results based on patients with relatively high and low risk by Kaplan–Meier survival curve. (**B**) The survival prediction based on the metabolism-based prognostic index by time-dependent ROC curve analysis.

Figure 6A,B shows the survival score and survival status of OSC patients in the low-risk and high-risk groups. Figure 6C shows the heatmap of the eight gene expression profiles in the TCGA dataset. The majority of the risk scores were <1.55; however, as the risk score increased, the survival time and the survival rate decreased. This metabolism-based prognostic indicator could serve as a significant tool for identifying patients with OSC based on latent discrete clinical outcomes (Figure 7A). The 1-, 3- and 5-year areas under the subject of the ROC curve were 0.653, 0.68 and 0.616, respectively, which indicated that a moderate incubation period could be used as a prognostic indicator of MRGs in survival monitoring (Figure 7B).

### Characteristics of hub MRGs

**Figure 8:**
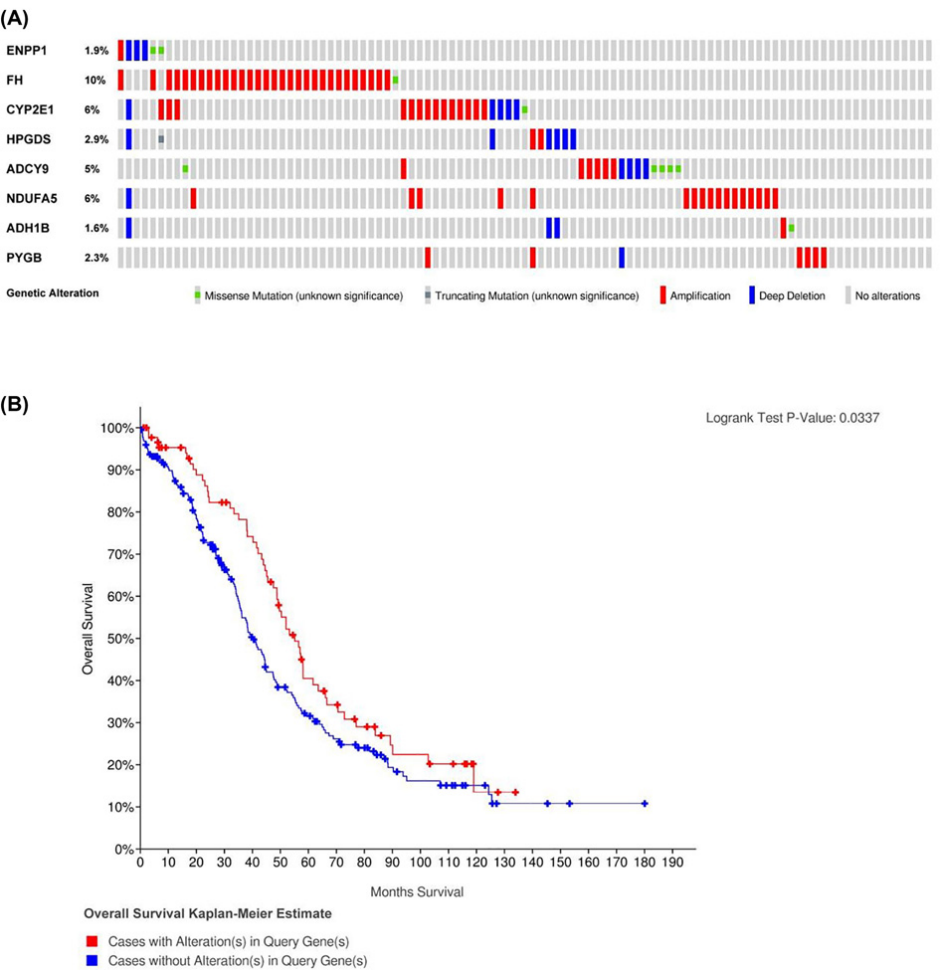
Mutation feature of hub MRGs (**A**) The mutation rate of FH, CYP2E1, ADCY9 and NDUFA5 was more than 5%. (**B**) OS Kaplan–Meier estimate based on patients with and without alterations in the cBioPortal for Cancer Genomics database (www.cbioportal.org).

Because these MRGs have important clinical value, we explored their molecular features comprehensively. We found that these genes were genetically altered, including changes in mRNA up-regulation and deep deletion (Figure 8A). Moreover, the Kaplan–Meier curves indicate significant differences in OS (*P*<0.05) for cases with alterations and cases without alterations in the eight genes, which indicated that the group with alterations was more closely correlated with poor prognosis than was another group without alterations (Figure 8B).

### GSEA

**Figure 9:**
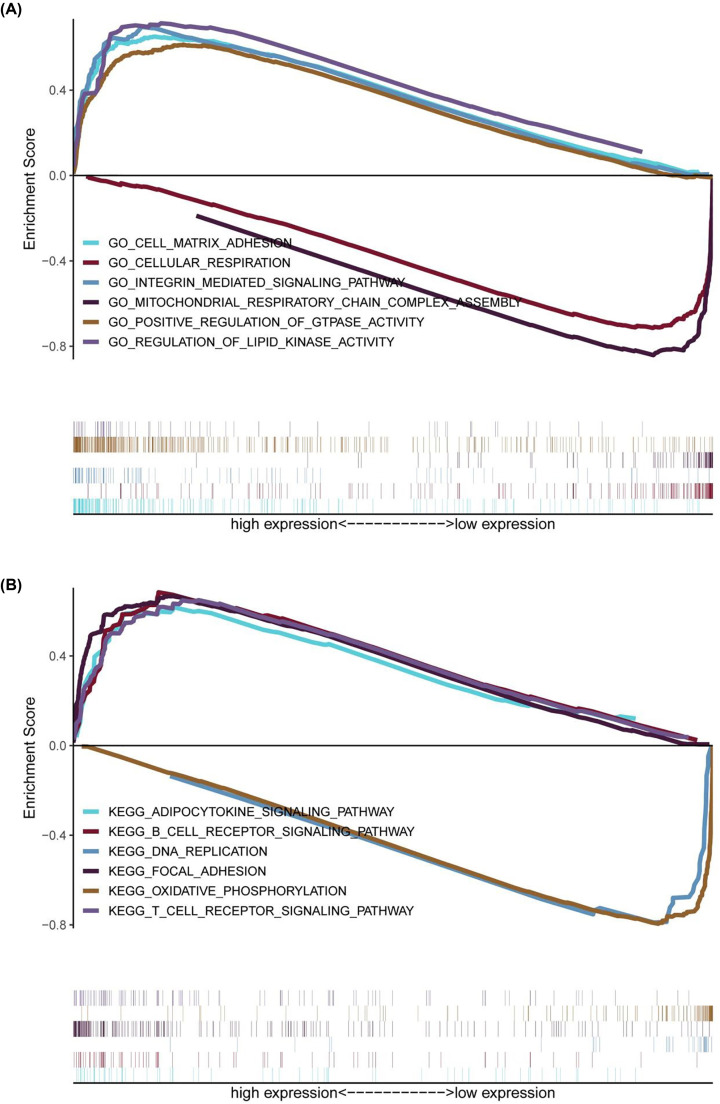
GSEA analysis (**A**) The six most obviously enriched BP GO terms. (**B**) The six most significantly enriched KEGG pathways.

GO-GSEA suggested that OSC samples were markedly enriched in energy metabolism-related biological processes, including ‘regulation of lipid kinase activity’ and ‘positive regulation of GTPase activity’. KEGG-GSEA indicated that OSC samples were clearly enriched in cellular metabolic pathways, including the adipocytokine signalling pathway and the oxidative phosphorylation pathway (Figure 9A,B).

### Verification of the drug sensitivity of hub MRG

GSCALite (http://bioinfo.life.hust.edu.cn/web/GSCALite/) [13], an important website for the cancer genome analysis platform, was utilised to perform the drug sensitivity analysis of eight hub MRGs in OSC. Moreover, we confirmed that the drug sensitivity of the hub MRG, PYGB, was significantly associated with chemotherapy resistance by the GSCALite database (Supplementary Figures S1 and S2). Therefore, these results indicated that PYGB might play a key role in the functions of these eight hub MRGs regarding the formation, development and progression of OSC.

### Ectopic expression of PYGB is correlated with clinical parameters in OSC

**Figure 10:**
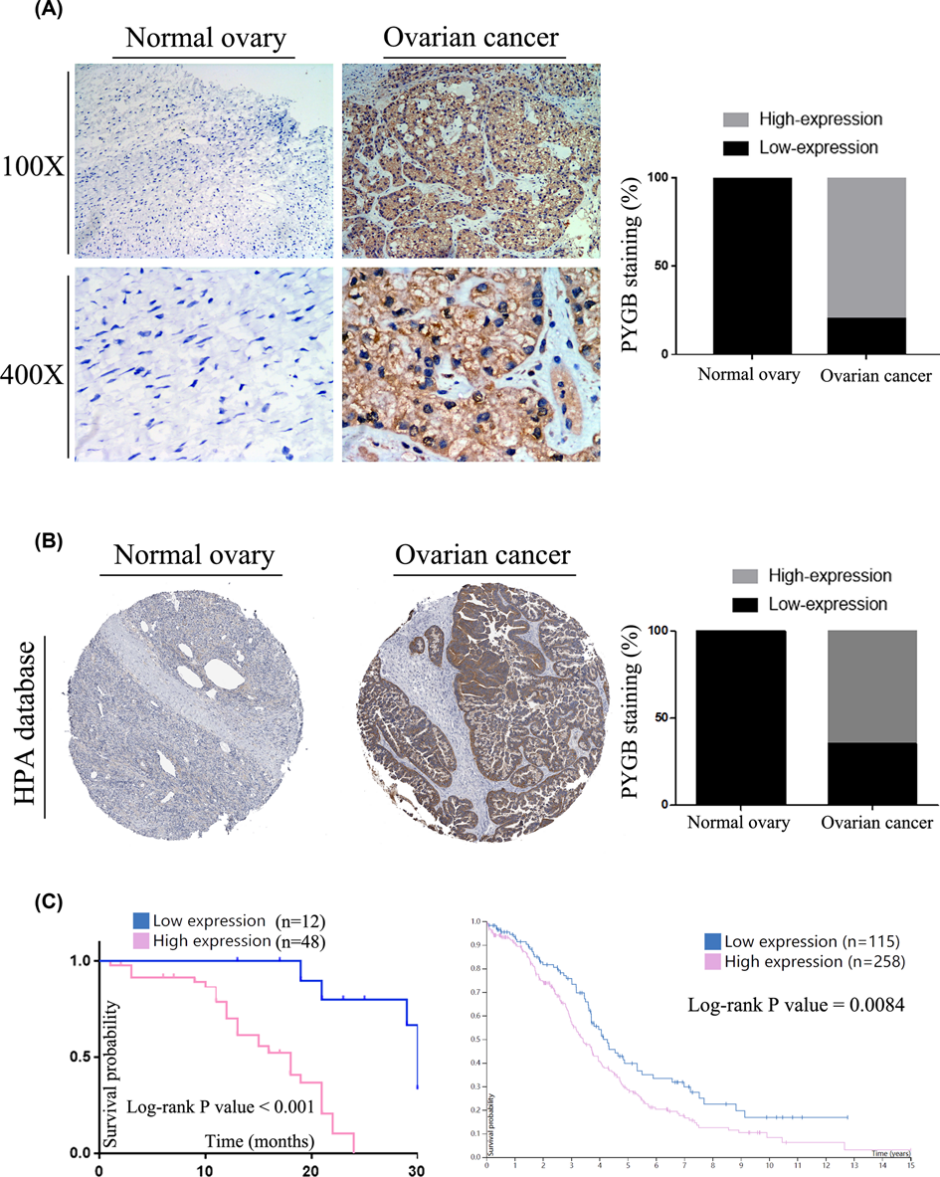
PYGB was overexpressed and correlated with prognosis in OC patients (**A**) IHC staining of PYGB expression in OC and normal ovarian tissue. PYGB showed strong cytoplasmic expression in OC compared with normal ovarian tissues. (**B**) IHC staining of PYGB in OC based on the HPA database. (**C**) OS Kaplan–Meier estimate based on patients with relatively high and low expression of PYGB (left is based on 60 OC patients; right is based on the HPA database.)

To further demonstrate the level of PYGB in OC, we confirmed the level of PYGB by IHC staining. As we predicted, the expression of PYGB is enhanced in the cancer tissues of 60 women with OC compared with normal ovarian tissues (Figure 10A). We used the HPA database to further verify the results, which indicated that PYGB was similarly increased in OC (Figure 10B).

The relationship between PYGB level and clinical parameters in OC was further analysed to confirm the pathophysiological significance of PYGB expression. Statistical analysis indicated that enhanced PYGB was positively correlated with some clinical parameters of OC (Table 1), such as diagnostic category (*P*<0.001), different FIGO stages (*P*=0.0203), absence or presence of metastasis (*P*=0.0304), and absence or presence of recurrence (*P*=0.0195), whereas we did not find significant correlations of PYGB expression with age, CA125 level, histological type, residual tumour after surgery, absence of macroscopic tumour or pathological grade in OC. Furthermore, our results and the information in the HPA database suggested that PYGB overexpression negatively correlated with the OS rate (Figure 10C, *P*<0.05), which indicated that PYGB played a key role in the development and progression of OC.

**Table 1:** The correlation between PYGB expression and clinicopathologic characteristics in IHC analysis

Variables	Number (*n*= 86)	PYGB expression		
		Low (%)	High (%)	*P*-value
Diagnostic category				<0.001
Normal	26	26	0	
Ovarian cancer	60	12	48	
Age (years)				0.7745
<55	43	9	34	
≥55	17	3	14	
CA 125 (IU/ml)				0.8675
≤500	11	2	9	
>500	49	10	39	
Histological type				
Histological type, High-grade serous	48	10	38	0.6708
Endometrioid	9	2	7	
Others	3	0	3	
FIGO stage				0.0203
I	4	1	3	
II	11	1	10	
III	28	2	26	
IV	17	8	11	
Residual tumour after surgery, no macroscopic tumuor (cm)				0.0856
≤1	17	1	16	
>1	43	11	32	
Pathological grade				0.2451
G1	6	0	6	
G2	10	1	9	
G3	44	11	33	
Lymph node metastases				0.0304
Yes	48	11	28	
No	22	1	20	
Recurrence				0.0195
Yes	33	3	30	
No	27	9	18	

Abbreviation: FIGO, the International Federation of Gynecology and Obstetrics.

## Discussion

OSC is one of the most common lethal malignancies worldwide. The slow rate of progress in the development of molecular targeted treatment and the absence of effective biomarkers for OSC prognostic monitoring make it necessary to further elucidate the molecular mechanisms leading to this condition. The exploration of metabolic mechanisms opens up an important perspective for OSC [1].

Although the importance of MRGs in cancer development has been well studied, to the best of our knowledge, no genome-wide analysis has been conducted to explore their clinical significance and molecular mechanism. In the present study, a personalised metabolic signature based on the selection and differential expression of MRGs is presented to measure cancer cell development and evaluate conditions of prognosis. This comprehensive and complete analysis of MRGs in OSC improves our understanding of their clinical implications and clarifies their underlying molecular features. The large number of OSC samples obtained through bioinformatic analysis in the present study helped us to obtain robust results.

In recent years, with the development of high-throughput sequencing technology, large databases, such as TCGA, SEER and GEO, have emerged, which provide an effective means for the selection of genetic markers. In the present study, we examined the expression profile of MRGs in TCGA to search for molecular markers to detect the prognosis of patients with OSC. We first screened 1466 differentially expressed MRGs in OSC and normal ovarian tissues. Considering that these genes may have a close association with the development and progression of OSC, we utilised functional analysis of these genes and showed that the KEGG pathway (metabolism-related pathway) was reduced. According to the abovementioned results, we speculated that tumour metabolism may play an important role in tumorigenesis. Glutamine, amino acids, glucose and free fatty acids are the basic and significant substances that support the growth and survival of cancer cells. These metabolites are either synthesised in cancer cells or are assimilated from the blood circulation [14]. In summary, a better understanding of the relationship between cell origin and metabolic status, as well as the function of MRGs, may facilitate efforts to better map the metabolic profile of OSCs.

When RWR was performed on the network, ACOT7, CERK, EHMT2, MTAP and PDE8A were also identified in association with the pathogenesis of OSC. In the network of these initial genes, it could interact with 200 metabolic genes considered the most closely related to the pathogenesis of ovarian cancer. On the basis of univariate Cox analysis, a total of eight genes were closely related to the prognosis of ovarian cancer patients: *ENPP1, FH, CYP2E1, HPGDS, ADCY9, NDUFA5, ADH1B* and *PYGB*. Further analysis helped us to distinguish high-risk and low-risk groups to develop the metabolism-based prognostic index. Furthermore, we extracted the expression profile, prognostic value and mutation status of this index to explore its possible clinical value, and we constructed an MRG network to reveal hub MRGs that regulate tumour metabolism and progression.

ENPP1, FH, CYP2E1, HPGDS, ADCY9, NDUFA5, ADH1B and PYGB were characterised in this network. Given the underlying mechanisms of these MRGs, studies on the functions and mechanisms of HPGDS, ADCY9 and NDUFA5 have not been reported in studies on OSC [15–19]. However, five of these eight hub MRGs have been studied, namely, ENPP1, FH, CYP2E1, ADH1B and PYGB. ENPP1 is increased in ovarian cancer and may promote migration [15]. A high level of FH could promote aggressive and metastatic behaviours [20]. CYP2E1 overexpression was correlated with inflammatory cytokines, including IL-6, IL-8, and TNF-α [21]. High expression of ADH1B was correlated with a markedly higher risk of residual disease in OSC [22], which played a significant role in accelerating ovarian cancer cell infiltration and may have enhanced the possibility of postoperative residual lesions [23]. PYGB clearly promoted ovarian cancer cell proliferation, invasion and migration via the wnt pathway [24]. Based on previous research, we could obtain only scarce information about the eight MRGs involved OSC patient survival. The oxidative phosphorylation pathway is the most important pathway in functional enrichment analysis, and it may be involved in the OSC process.

Currently, some of the prognostic characteristics of cancer have been elucidated with the help of large public databases. For example, Bao et al. successfully obtained a 4-lncRNA signature by analysing RNA-Seq data from 234 BC patients from the TCGA database, which has prognostic value [25]. Nevertheless, these studies only focussed on classic tumour biological behaviour and ignored tumour metabolism. We attach considerable importance to the classic biological behaviour as well as tumour metabolism. Therefore, prognostic characteristics are expected to act as applications for clinical medicine. Nevertheless, the limitations of the present study lie in its retrospective features. Owing to the limited number of cases, the present study was unable to detect ENPP1, FH, CYP2E1, HPGDS, ADCY9, NDUFA5, ADH1B and PYGB expression in OSC and normal ovarian tissues. Moreover, PYGB was involved in drug sensitivity and increased in OSC identified by IHC, which could indicate a poor prognosis for OSC patients. However, the molecular mechanism underlying the development and progression of OC has not been fully elucidated.

As we look to the future, there are still numerous problems to solve. For instance, the relationship among metabolomics, immune genomics, proteomics and epigenomics should be investigated to further describe global metabolic alterations in OSC. It is also important to further explore the potential relationship between metabolomics disorders and precancerous lesions. The prognostic feature may have important clinical instructive significance. Our findings may provide new ideas for the individual treatment of OSC.

## Conclusion

In the present study, a comprehensive analysis of the MRG expression profile and corresponding prognostic data identified eight prognostic MRGs (i.e., ENPP1, FH, CYP2E1, HPGDS, ADCY9, NDUFA5, ADH1B and PYGB). The discovery of genes in the metabolic pathway also opens up new possibilities for the treatment of ovarian cancer. Combined with molecular expression and survival analysis, we constructed a new metabolism-based prognostic index risk score model that can better predict the survival of OSC patients. In addition, we found that PYGB played a key role in drug sensitivity, and it was increased in OC compared with normal ovarian tissues, which also indicates an unfavourable prognosis in OC patients. Nevertheless, further prospective trials are warranted to verify the clinical efficacy of these results and contribute to the best therapy.

## Supplementary Material

Supplementary Figures S1 and S2

Supplementary Data

## Abbreviations

BC: breast cancer
FPKM: fragments per kilobase per million
GEO: Gene Expression Omnibus
GO: gene ontology
GSEA: gene set enrichment analysis
GTEX: Genotype Tissue Expression
HPA: Human Protein Atlas
IHC: immunohistochemistry
KEGG: Kyoto Encyclopedia of Genes and Genomes
MRG: metabolism-related gene
OC: ovarian cancer
OS: overall survival
OSC: ovarian serous cystadenocarcinom
ROC: receiver operating characteristic
RWR: random walking with restart
SEER: Surveillance, Epidemiology, and End Results
TCGA: The Cancer Genome Atlas
