# Supplementary material for: Identification and validation of a prognostic index based on a metabolic-genomic landscape analysis of ovarian cancer
Source: Biosci Rep. 2020 Sep 28;40(9):BSR20201937. doi: 10.1042/BSR20201937 (PMC7527429; doi:10.1042/BSR20201937)
Supplement: Supplementary Figures S1 and S2 [file BSR-2020-1937_supp.pdf]

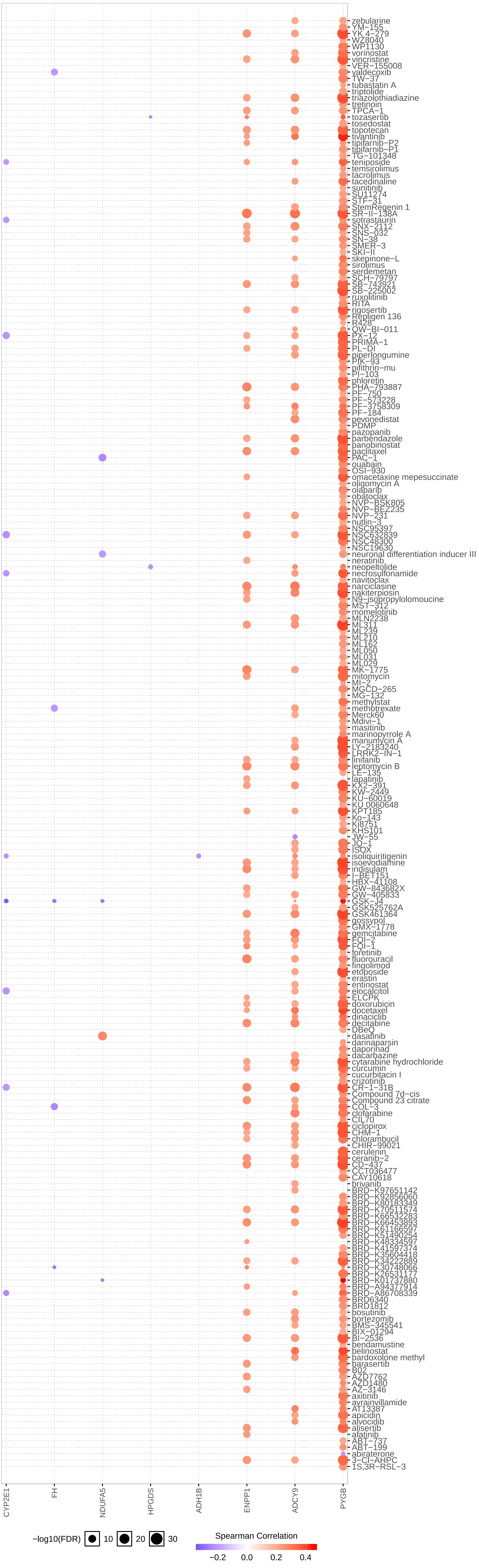
zebularine
YM−155
YK 4−279
WZ8040
WP1130
vorinostat
vincristine
VER−155008
valdecoxib
TW−37
tubastatin A
triptolide
triazolothiadiazine
tretinoin
TPCA−1
tozasertib
tosedostat
topotecan
tivantinib
tipifarnib−P2
tipifarnib−P1
TG−101348
teniposide
temsirolimus
tacrolimus
tacedinaline
sunitinib
SU11274
STF−31
StemRegenin 1
SR−II−138A
sotrastaurin
SNX−2112
SNS−032
SN−38
SMER−3
SKI−II
skepinone−L
sirolimus
serdemetan
SCH−79797
SB−743921
SB−225002
ruxolitinib
RITA
rigosertib
Repligen 136
R428
QW−BI−011
PX−12
PRIMA−1
PL−DI
piperlongumine
PFK−93
pifithrin−mu
PI−103
phloretin
PHA−793887
PF−750
PF−573228
PF−3758309
PF−184
pevonedistat
PDMP
pazopanib
parbendazole
panobinostat
paclitaxel
PAC−1
ouabain
OSI−930
omacetaxine mepesuccinate
oligomycin A
olaparib
obatoclax
NVP−BSK805
NVP−BEZ235
NVP−231
nutlin−3
NSC95397
NSC632839
NSC48300
NSC19630
neuronal differentiation inducer III
neratinib
neopeltolide
necrosulfonamide
navitoclax
narciclasine
nakiterpiosin
N9−isopropylolomoucine
MST−312
momelotinib
MLN2238
ML311
ML239
ML210
ML162
ML050
ML031
ML029
MK−1775
mitomycin
MI−2
MGCD−265
MG−132
methylstat
methotrexate
Merck60
Mdivi−1
masitinib
marinopyrrole A
manumycin A
LY−2183240
LRRK2−IN−1
linifanib
leptomycin B
LE−135
lapatinib
KX2−391
KW−2449
KU−60019
KU 0060648
KPT185
Ko−143
Ki8751
KHS101
JW−55
JQ−1
ISOX
isoliquiritigenin
isoevodiamine
indisulam
I−BET151
HBX−41108
GW−843682X
GW−405833
GSK−J4
GSK525762A
GSK461364
gossypol
GMX−1778
gemcitabine
FQI−2
FQI−1
foretinib
fluorouracil
fingolimod
etoposide
erastin
entinostat
elocalcitol
ELCPK
doxorubicin
docetaxel
dinaciclib
decitabine
DBeQ
dasatinib
darinaparsin
daporinad
dacarbazine
cytarabine hydrochloride
curcumin
cucurbitacin I
crizotinib
CR−1−31B
Compound 7d−cis
Compound 23 citrate
COL−3
clofarabine
CIL70
ciclopirox
CHM−1
chlorambucil
CHIR−99021
cerulenin
ceranib−2
CD−437
CCT036477
CAY10618
brivanib
BRD−K97651142
BRD−K92856060
BRD−K80183349
BRD−K70511574
BRD−K66532283
BRD−K66453893
BRD−K61166597
BRD−K51490254
BRD−K48334597
BRD−K41597374
BRD−K35604418
BRD−K34222889
BRD−K30748066
BRD−K26531177
BRD−K01737880
BRD−A94377914
BRD−A86708339
BRD6340
BRD1812
bosutinib
bortezomib
BMS−345541
BIX−01294
BI−2536
bendamustine
belinostat
bardoxolone methyl
barasertib
B02
AZD7762
AZD1480
AZ−3146
axitinib
avrainvillamide
AT13387
apicidin
alvocidib
alisertib
afatinib
ABT−737
ABT−199
abiraterone
3−Cl−AHPC
1S,3R−RSL−3
CYP2E1
FH
NDUFA5
HPGDS
ADH1B
ENPP1
ADCY9
PYGB
−log10(FDR)
10
20
30
Spearman Correlation
−0.2
0.0
0.2
0.4

Supplementary Figure S1. CTRP drug sensitivity

The CTRP drug sensitivity was analysed in theGSCALitedatabase for these eight genes.

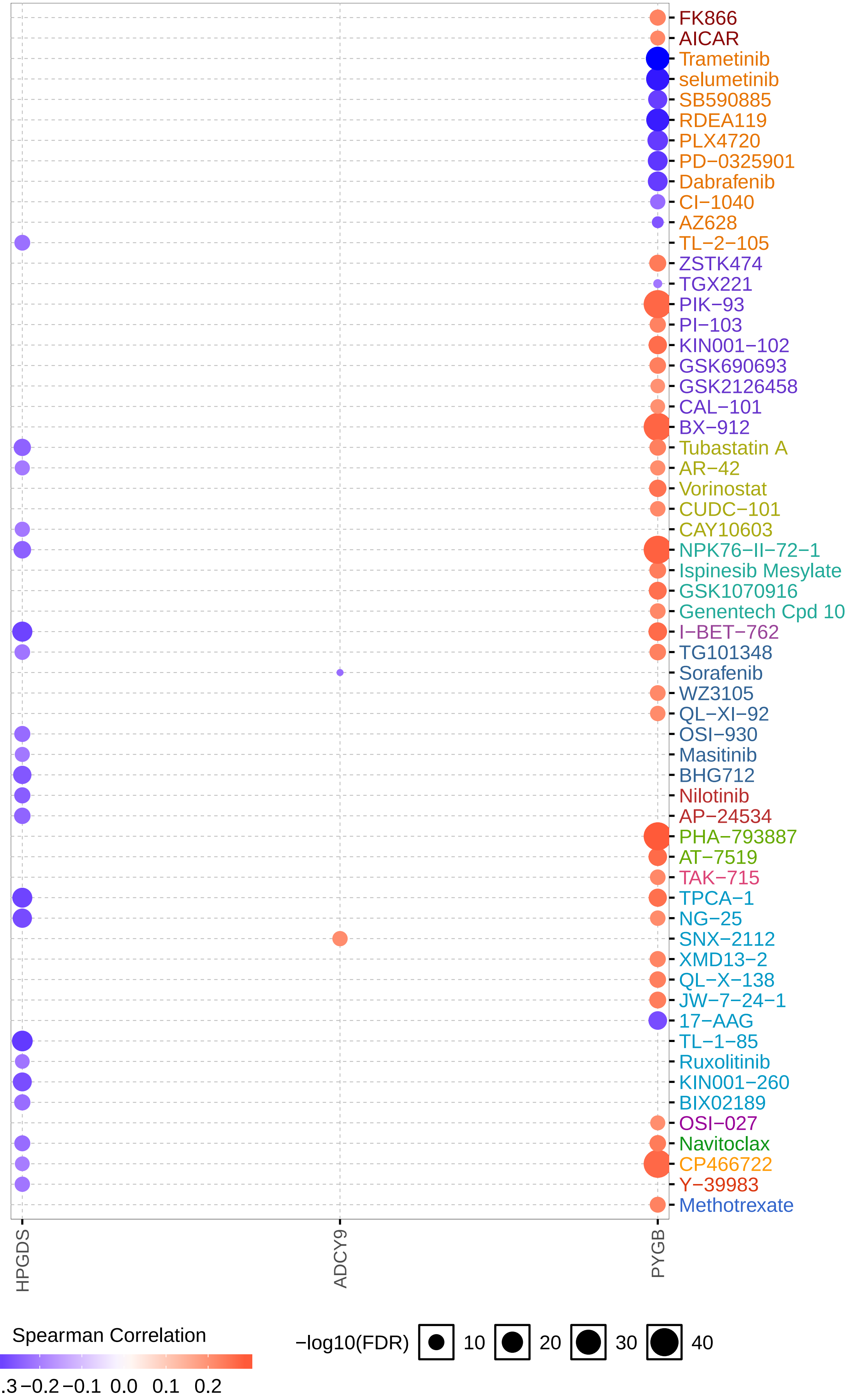
FK866
AICAR
Trametinib
selumetinib
SB590885
RDEA119
PLX4720
PD−0325901
Dabrafenib
CI−1040
AZ628
TL−2−105
ZSTK474
TGX221
PIK−93
PI−103
KIN001−102
GSK690693
GSK2126458
CAL−101
BX−912
Tubastatin A
AR−42
Vorinostat
CUDC−101
CAY10603
NPK76−II−72−1
Ispinesib Mesylate
GSK1070916
Genentech Cpd 10
I−BET−762
TG101348
Sorafenib
WZ3105
QL−XI−92
OSI−930
Masitinib
BHG712
Nilotinib
AP−24534
PHA−793887
AT−7519
TAK−715
TPCA−1
NG−25
SNX−2112
XMD13−2
QL−X−138
JW−7−24−1
17−AAG
TL−1−85
Ruxolitinib
KIN001−260
BIX02189
OSI−027
Navitoclax
CP466722
Y−39983
Methotrexate
HPGDS
ADCY9
PYGB
Spearman Correlation
.3 −0.2 −0.1 0.0 0.1 0.2
−log10(FDR)
10
20
30
40

Supplementary Figure S2. GDSC drug sensitivity
The GDSC drug sensitivity was analysed in the GSCA Lite database for these eight genes.
